# Urban Market Gardening and Rodent-Borne Pathogenic *Leptospira* in Arid Zones: A Case Study in Niamey, Niger

**DOI:** 10.1371/journal.pntd.0004097

**Published:** 2015-10-05

**Authors:** Gauthier Dobigny, Madougou Garba, Caroline Tatard, Anne Loiseau, Max Galan, Ibrahima Kadaouré, Jean-Pierre Rossi, Mathieu Picardeau, Eric Bertherat

**Affiliations:** 1 Institut de Recherche pour le Développement, Centre de Biologie pour la Gestion des Populations (UMR IRD-INRA-CIRAD-SupAgro Montpellier), Campus International de Baillarguet CS30016, Montferrier-sur-Lez, France; 2 Direction Générale de la Protection des Végétaux, Ministère de l’Agriculture, Niamey, Niger; 3 Institut National de la Recherche Agronomique, CBGP, Campus International de Baillarguet CS30016, Montferrier-sur-Lez, France; 4 Centre Régional Agrhymet, USAid/Fews-Net, Niamey, Niger; 5 Institut Pasteur, Unité de Biologie des Spirochètes, Centre National de Référence et Centre Collaborateur de l’OMS de la Leptospirose, Paris, France; 6 World Health Organization, Geneva, Switzerland; University of California San Diego School of Medicine, UNITED STATES

## Abstract

Leptospirosis essentially affects human following contact with rodent urine-contaminated water. As such, it was mainly found associated with rice culture, recreational activities and flooding. This is also the reason why it has mainly been investigated in temperate as well as warm and humid regions, while arid zones have been only very occasionally monitored for this disease. In particular, data for West African countries are extremely scarce. Here, we took advantage of an extensive survey of urban rodents in Niamey, Niger, in order to look for rodent-borne pathogenic *Leptospira* species presence and distribution across the city. To do so, we used high throughput bacterial 16S-based metabarcoding, *lipL32* gene-targeting RT-PCR, rrs gene sequencing and VNTR typing as well as GIS-based multivariate spatial analysis. Our results show that leptospires seem absent from the core city where usual *Leptospira* reservoir rodent species (namely *R*. *rattus* and *M*. *natalensis*) are yet abundant. On the contrary, *L*. *kirschneri* was detected in *Arvicanthis niloticus* and *Cricetomys gambianus*, two rodent species that are restricted to irrigated cultures within the city. Moreover, the VNTR profiles showed that rodent-borne leptospires in Niamey belong to previously undescribed serovars. Altogether, our study points towards the importance of market gardening in maintain and circulation of leptospirosis within Sahelian cities. In Africa, irrigated urban agriculture constitutes a pivotal source of food supply, especially in the context of the ongoing extensive urbanization of the continent. With this in mind, we speculate that leptospirosis may represent a zoonotic disease of concern also in arid regions that would deserve to be more rigorously surveyed, especially in urban agricultural settings.

## Author Summary

We surveyed rodent-borne *Leptospira* in rodents from Niamey, the capital town of Niger, using bacterial metabarcoding, RT-PCR, sequencing, VNTR typing and GIS-based geostatistics. Two new serovars of *Leptospira kirschneri* were identified in *Arvicanthis niloticus* and *Cricetomys gambianus*, two species that inhabit exclusively urban irrigated gardens. Since no rodent-borne leptospires could be found in the core city, our results point towards the importance of urban agriculture in the maintaining and the circulation of these bacteria in cities from semi-arid regions where they are usually poorly documented and even hardly looked for. Accordingly, this is one of the very rare mentions of these zoonotic agents in Sahel, and the first one in Niger. Keeping in mind the critical role of urban gardening for food security in extensively growing West African cities, we believe that leptospirosis should be more closely scrutinized in Sahelian countries where numerous cases of human fevers are of unknown origin.

## Introduction

*Leptospira* is a genus of spirochetes which comprises three lineages, one of which grouping pathogenic species for both animal and human [1]. Leptospirosis is a major zoonotic disease that may affect at least 500,000 and potentially up to 1 million persons, and kill ~60,000 ones per year worldwide [2–5]. Its incidence remains poorly documented because leptospirosis leads to clinical signs that are difficult to distinguish from other widespread endemic pathologies such as dengue, malaria, influenza, etc. [6]. In addition, many countries where it has an obvious burden lack appropriate diagnostic facilities, thus strongly suggesting that cases may be massively underreported [2, 4].

Among other mammals, rodents, especially rats, constitute major reservoirs of *Leptospira* spp.: the bacterium resides in the host renal tubules and is then excreted into the environment through its urine. Leptospirosis is thought to be essentially associated with water where humans get contaminated following contact with the pathogen through skin abrasions or mucous membranes (reviews in [4, 7]). In particular, rice culture, recreational water activities and flooding have been massively linked to leptospirosis. This is the reason why the disease was essentially looked for, and found in temperate as well as warm and humid tropical regions (reviewed in [8]). Surveys in arid zones are rare, although some mentions exist from desert to sub-desert areas (e.g., Somalia: [9]; Arizona: [10]; Mexico: [11]; Brazil: [12]), thus suggesting that *Leptospira* may be much more widespread than currently thought and could also extend to dry regions. As an example, prevalence in wild Malagasy mammals was found higher in Northern areas of the island where rainfalls are weaker [13].

Mentions of *Leptospira* in Africa (review in [14–16]) are quite scattered, and even very rare for some particular regions (see Fig. 2 in [14]). For instance, in the West African Sahel zone, some data are available for Senegal (two investigations in both humans and cattle in the 1970s), Chad (one report in a dog in 2008) and Mali (one human case report in the 1990s, and one investigation in cattle in the early 1970s) while no monitoring has ever been conducted in Burkina-Faso or Niger [14]. Yet, reported sporadic epidemics in various parts of the continent reflect a lack of knowledge of the disease rather than a truly narrow distribution of *Leptospira* [8]. This suggests that further investigations in Africa in general, and in arid zones in particular are required. Moreover, leptospirosis is often associated with disadvantaged urban areas where poor sanitation together with elevated rodent-human interactions increase the risk of rodent-to-human transmission (e.g., [17–19]; reviewed in [4]). Taking into account the impressive growth of African cities [20], there is little doubt that leptospirosis will be a major (re)emerging disease on the continent [14].

Niger, focus of the present study, ranks last of the World for the Human Development Index (187 out of 187; [21]). The capital city, Niamey, lies on the Niger River in the western part of the country, and is located in the typical Sahelian bioclimatic zone. As such, it is characterized by high temperatures (monthly average temperatures between 22–36°C) and low rainfalls (~540 mm per year) with a single rainy season between May and September. It was created *ex nihilo* at the very end of the nineteenth century by French colonizers (reviewed in [22]). During the last decades, the city has been experiencing an explosive spatial and demographic growth with its population increasing from >30,000 in the late 1950s, to 707,000 in 2001, and currently reaching more than 1,000,000 inhabitants [22–24]. As often in such cases, this rapid urbanization is characterized by many informal settlements and insufficient sanitary services. Accordingly, data hence knowledge about zoonotic pathogens that may circulate in Niger are extremely scarce, potentially explaining why so many fevers are misdiagnosed as malaria (i.e., more than 55% in the rainy season, and up to 95% during the dry season; [25]). Expectedly, *Leptospira* appears among the top candidate pathogens that may explain these so many fevers of unknown origin [26].

These are the reasons why we took advantage of a monitoring of urban rodents conducted in Niamey, the main town of Niger [27], to perform the first survey of rodent-borne *Leptospira* in this very poor country where zoonoses are dramatically under-documented.

## Materials and Methods

### Ethics statements

The whole rodent trapping campaign was validated by national and local authorities (scientific partnership agreement number 301027/00 between IRD and the Republic of Niger). At the French level, all sampling procedures were approved by the “Comité d’Ethique pour l’Expérimentation Animale—Languedoc Roussillon” (agreement number C34-169-1, valid until 25th July 2017) and were conducted by biologists from the CBGP holding certificates to carry out experiments on live animals (agreement number C34-488). None of the rodent species investigated in the present study has protected status (see UICN and CITES lists). All animals were treated in a humane manner in accordance with guidelines of the American Society of Mammalogists. All rodents were euthanized through cervical dislocation. Permit to enter and work within private properties were systematically obtained through oral but explicit agreement from adequate institutional (research agreement quoted above; mayor) and traditional authorities (both neighborhood and family chiefs).

### Rodent sampling and species-specific identifications

**Fig 1:**
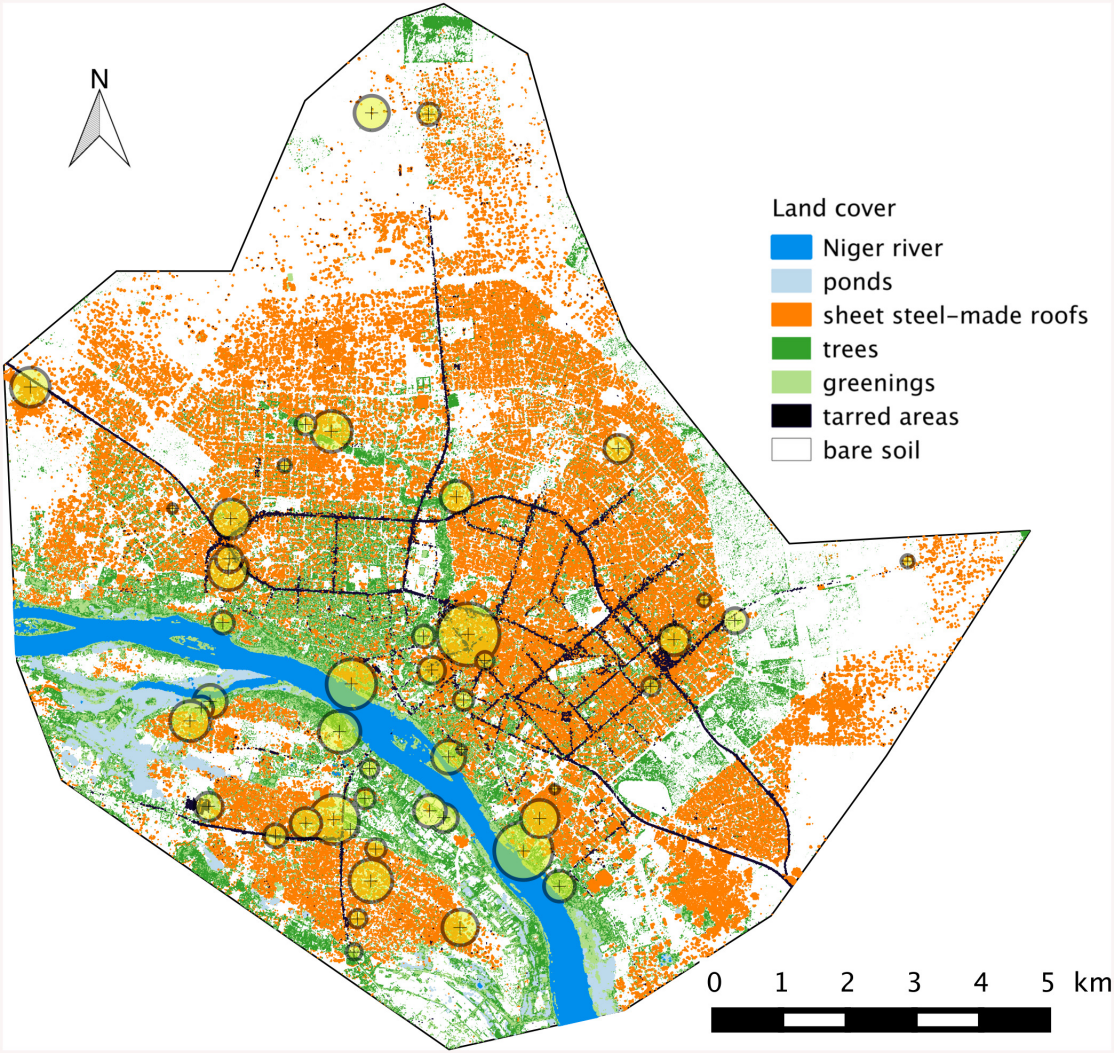
Map of localities within Niamey where rodents were trapped and screened for the presence / absence of *Leptospira*. The background corresponds to a GIS view of the city, with seven land cover categories taken into account (see text for details). Circle sizes is proportional to sample size at each sampling locality.

From October 2009 to February 2011, an extensive survey of urban rodent assemblages was conducted in 52 localities of Niamey, Niger, thus allowing the exploration of more than 215 trapping sites with an effort of >14,500 night-traps (see details in [27]). Among the 987 rodents captured, 578 were included in the present screening of *Leptospira*. They consisted in 66 *Arvicanthis niloticus*, 12 *Cricetomys gambianus*, 350 *Mastomys natalensis*, 50 *Mus musculus* and 100 *Rattus rattus* originating from 49 localities sites within the city (Table 1 and Fig 1).

**Table 1:** Sample used in the present study. Within-city localities (“locality”), habitats (“habitat”) and rodent species (“species”) that were investigated for rodent-borne *Leptospira* using various molecular techniques (“technique”) are provided. “NGS”, “RT-PCR”, “sequencing” and “VNTR” stand for 16S metabarcoding, *lipL32*-centred RT-PCR, *rrs* gene sequencing and VNTR profile typing, respectively. Presence/absence of *Leptospira* (“Lepto”) is also indicated (i.e., “yes” or “no”, respectively). Numbers inside brackets correspond to rodent sample size.

Locality	Habitat	Species (N)	Technique				Lepto	GPS	
			NGS	RT-PCR	Sequencing	VNTR		Lat (N)	Long (E)
ABA	factory	*R*. *rattus* (43)	+ (43)	-	-	-	no	13.4895	2.12275
BAF2	households	*M*. *natalensis* (10)	+ (10)	-	-	-	no	13.54401	2.1357
BOU	households	*M*. *natalensis* (13)	+ (13)	-	-	-	no	13.53742	2.11331
CGA	households	*R*. *rattus* (14)	+ (14)	-	-	-	no	13.50222	2.11235
COA	households	*M*. *natalensis* (1)	+ (1)	-	-	-	no	13.53571	2.07399
CRA-1	garden	*C*. *gambianus* (3)	-	+ (3)	-	-	no	13.49235	2.09877
CRA-2	garden	*C*. *gambianus* (5)	-	+ (5)	+ (1)	+ (1)	yes (1)	13.49655	2.10079
CRA-3	garden	*A*. *niloticus* (4)	+ (4)	+ (4)	-	-	no	13.5006	2.10141
CYA	households	*M*. *natalensis* (28)	+ (28)	-	-	-	no	13.51204	2.09884
	households	*R*. *rattus* (3)	+ (3)	-	-	-	no		
DAR	households	*M*. *natalensis* (21)	+ (21)	-	-	-	no	13.54624	2.09594
GAM	households	*M*. *natalensis* (18)	+ (18)	-	-	-	no	13.49392	2.12501
GAM-1	households	*M*. *natalensis* (1)	+ (1)	-	-	-	no	13.49792	2.12705
GAW	households	*M*. *natalensis* (5)	+ (5)	-	-	-	no	13.4897	2.10232
GNA	households	*M*. *natalensis* (16)	+ (16)	-	-	-	no	13.47908	2.11402
GOU	households	*M*. *musculus* (6)	+ (6)	-	-	-	no	13.51856	2.10883
GRM	households	*M*. *musculus* (43)	+ (43)	-	-	-	no	13.51882	2.115
	households	*R*. *rattus* (6)	+ (6)	-	-	-	no		
GRM-M	market	*R*. *rattus* (4)	+ (4)	-	-	-	no	13.51527	2.11732
HPO	households	*M*. *musculus* (1)	+ (1)	-	-	-	no	13.50992	2.11438
	households	*R*. *rattus* (4)	+ (4)	-	-	-	no		
J-CYA	garden	*A*. *niloticus* (1)	-	+ (1)	-	-	no	13.52029	2.08104
	households	*R*. *rattus* (5)	+ (5)	-	-	-	no		
J-DAR	garden	*A*. *niloticus* (5)	+ (3)	+ (5)	-	-	no	13.54714	2.09238
J-GAM	garden	*A*. *niloticus* (11)	+ (10)	+ (11)	+ (4)	+ (4)	yes (5)	13.48473	2.12775
	households	*M*. *natalensis* (1)	+ (1)	-	-	-	no		
J-KIR-1	garden	*A*. *niloticus* (4)	+ (1)	+ (4)	+ (1)	+ (1)	yes (1)	13.49397	2.1117
	households	*M*. *natalensis* (6)	+ (6)	-	-	-	no		
J-KIR-2	households	*M*. *natalensis* (1)	+ (1)	-	-	-	no	13.47573	2.09936
	garden	*C*. *gambianus* (2)	-	+ (2)	-	-	no		
J-LMO-1	garden	*A*. *niloticus* (12)	+ (12)	+ (12)	-	-	no	13.50962	2.0793
	garden	*C*. *gambianus* (2)	-	+ (2)	-	-	no		
J-LMO-2	garden	*A*. *niloticus* (7)	+ (5)	+ (7)	+ (2)	+ (2)	yes (2)	13.50871	2.07808
J-NOG	garden	*A*. *niloticus* (22)	+ (15)	+ (22)	-	-	no	13.50558	2.09723
KAR	households	*M*. *natalensis* (31)	+ (31)	-	-	-	no	13.49366	2.0965
KAR-1	households	*M*. *natalensis* (7)	+ (7)	-	-	-	no	13.49143	2.08843
KAR-2	households	*M*. *natalensis* (12)	+ (12)					13.49316	2.09262
KIR	factory	*R*. *rattus* (13)	+ (13)	-	-	-	no	13.49489	2.10978
KIR-1	households	*M*. *natalensis* (4)	+ (4)	-	-	-	no	13.48022	2.09984
KOT	households	*M*. *natalensis* (6)	+ (6)	-	-	-	no	13.58922	2.10928
KOU	households	*M*. *natalensis* (19)	+ (19)	-	-	-	no	13.55207	2.05424
KOU-1	households	*M*. *natalensis* (2)	+ (2)	-	-	-	no	13.56106	2.04155
LMO	households	*M*. *natalensis* (20)	+ (20)	-	-	-	no	13.50696	2.07653
PEM	market	*R*. *rattus* (3)	+ (3)	-	-	-	no	13.51396	2.10997
	market	*M*. *natalensis* (6)	+ (6)	-	-	-	no		
PKE	households	*M*. *natalensis* (23)	+ (23)	-	-	-	no	13.48536	2.10164
REC	households	*M*. *natalensis* (2)	+ (2)	-	-	-	no	13.54157	2.0895
RFN	households	*M*. *natalensis* (2)	+ (2)	-	-	-	no	13.52893	2.17587
ROF	households	*M*. *natalensis* (8)	+ (8)	-	-	-	no	13.52081	2.15193
ROF-1	households	*M*. *natalensis* (2)	+ (2)	-	-	-	no	13.52358	2.14766
RTO	factory	*M*. *natalensis* (10)	+ (10)	-	-	-	no	13.49539	2.07916
TCH	households	*M*. *natalensis* (15)	+ (15)	-	-	-	no	13.58936	2.10137
TER	households	*R*. *rattus* (1)	+ (1)	-	-	-	no	13.50323	2.11413
WAD	households	*M*. *natalensis* (12)	+ (12)	-	-	-	no	13.5182	2.14351
WAD-1	coach station	*R*. *rattus* (4)	+ (4)	-	-	-	no	13.51186	2.14032
YAB	households	*M*. *natalensis* (20)	+ (20)	-	-	-	no	13.5274	2.08175
YAB-1	households	*M*. *natalensis* (9)	+ (9)	-	-	-	no	13.52891	2.08186
YAH	households	*M*. *natalensis* (19)	+ (19)	-	-	-	no	13.53435	2.08208

African rodent species identification may sometimes be difficult due to the frequent co-occurrence of sibling taxa, notably in the genera *Rattus* [28], *Arvicanthis* and *Mastomys* [29]. This is the reason why a special attention was paid to taxonomic diagnosis which relied on karyotyping (for *Arvicanthis*, *Mus* and *Mastomys*), cytochrome b gene sequencing (for *Arvicanthis* and *Rattus*), PCR and species-specific RFLP (for *Mastomys*) and genotyping (for *Mastomys* and *Rattus*). All these procedures have been described in details elsewhere (see [27], and references therein).

### 16S gene-based metabarcoding of bacteriomes of rodent pools

Individual genomic DNA was extracted from ethanol-preserved kidney tissue using the Qiagen DNeasy Blood and Tissue Kit, and was quantified using Nanodrop technology (Thermoscientific). Kidney DNA samples were then prepared in equimolar concentration. Pools grouping 50 rodent individual DNA samples each were then arranged by species as follows: (i) one pool made of 50 *A*. *niloticus* from 7 localities, (ii) one pool of 50 *M*. *musculus* from 3 localities, (iii) two pools with 50 black rats each from 11 localities, respectively, and (iv) seven pools of 50 *M*. *natalensis* each and representing 32 localities. Samples were chosen in order to cover most (when not all) localities where each species had been found during a recent broader survey of urban rodents of Niamey (Table 1; see [27]). The eleven pools of DNA were then screened for the presence of bacteria using universal PCR primers targeting the hypervariable region V4 of the 16S rRNA gene (251bp) via Illumina MiSeq (Illumina) high throughput sequencing. The V4 region has been proven to offer excellent taxonomic resolution for bacteria at the genus level [30]. A multiplexing strategy enabled the identification of bacterial genera in each pool sample. We followed the method detailed in Kozich et al. [31] for PCR amplification, indexing, pooling of PCR products and de-multiplexing. Bacteria taxonomic identifications at the generic level were performed using the Silva SSU Ref NR 119 database (http://www.arb-silva.de/projects/ssu-ref-nr/) as a reference [32]. Each DNA pool was analyzed in triplicate using three independent PCRs and three amplicon libraries in the same next generation sequencing (NGS) run using a MiSeq sequencer (Illumina).

### RT-PCR screening of *lipL32* gene in individual rodents from positive pools

Rodents that belonged to metabarcoding *Leptospira*-positive pools as well as 16 *A*. *niloticus* and 12 *C*. *gambianus* which had not been included in the latter NGS-based survey were all individually screened for pathogenic *Leptospira* species using a dedicated Real Time PCR-based test.

To do so, sequences of *lipL32* gene from *Leptospira kirschneri* (AF121192), *L*. *interrogans* (AF181553, AF245281, AF366366, LIU89708), *L*. *borgpetersenii* (AF181554), *L*. *santarosai* (AF181555) and *L*. *noguchii* (AF181556) were aligned, and a consensus sequence was determined using BioEdit v.7.1.9. New forward (LIP32BF: 5’-AGC TCT TTT GTT CTG AGC GA-3’) and reverse (LIP32BR: 5’-TAC GAA CTC CCA TTT CAG CGA TTA-3’) primers were designed from this consensus sequence using the Light Cycler Probe design software v.2.0 (Roche). This new set of primers was proved to detect most known pathogenic *Leptospira* species (namely *L*. *interrogans*, *L*. *borgpetersenii* and *L*. *kirschneri* in ‘wet lab’, as well as *L*. *santarosai* and *L*. *noguchii in silico*) with lower Ct values than the primers used in recent *lipL32* RT-PCR-based survey (e.g., [33, 34]). We used the TaqMan probe (FAM-5′-AAA GCC AGG ACA AGC GCC G-3′-BHQ1) previously described in Stoddard et al. [33], thus allowing us to amplify a 199 pb-long fragment of the leptospiral *lipL32* gene.

RT-PCR reactions were performed using a LightCycler 480 (Roche) in 96-well microtitre plates with 10μL as final volume for each reaction. Optimal amplification conditions were obtained with 0.5μM of each primer, 0.2μM of probe, 2X of Probe Master buffer (Roche), 0.5U of Fast start Taq DNA polymerase (Roche) and 2μL of sample DNA. RT-PCR program consisted in an initial denaturing step at 95°C for 10 min, followed by 50 cycles of 95°C for 15s, 60°C for 30s and 72°C for 1s, and a final cooling step to 40°C. All samples were investigated in independent duplicates. Genomic DNA isolated from *L*. *interrogans* serovar Canicola and *L*. *borgpetersenii* serovar Tarassovi were used as positive controls. The Beta Actin gene was amplified from all samples as an internal RT-PCR control in order to detect false negative results [35].

### Identification of *Leptospira* species and subspecies through partial *16S rRNA* sequencing and VNTR profiles

A 330 pb-long fragment of the *rrs* gene was amplified from genomic DNA of the RT-PCR-positive rodents. Primers A and B were used for a first amplification; when the PCR was negative, a nested PCR was performed with primers C and D [36]. PCR products were sequenced in both directions at Eurofins Genomics. Species-specific identification was performed through Blastn (option Megablast for highly similar sequences) procedure under NCBI database.

Identification at the subspecies level was performed by multiple-locus variable-number tandem repeat analysis (MLVA) using the loci VNTR4, VNTR7 and VNTR10 as previously described [37] with the following modifications. MLVA was performed on DNA extracts using 70 cycles of amplification with a higher concentration of Taq polymerase (GE Healthcare). The sizes of the amplified products were then analysed using a 1% agarose gel electrophoresis, and the profiles were compared with the database of the National Reference Center for Leptospirosis (Institut Pasteur, Paris, France).

### GIS-based modeling of the distribution of suitable areas for rodent-borne *Leptospira*

Our purpose here was to map the most suitable within-city areas for *Leptospira*-carrying rodent species, as identified by molecular methods. Since reservoir rodents in Niamey were all found to belong to rural-like species (i.e. *A*. *niloticus* and *C*. *gambianus*; see below) and since these latter species strictly segregate spatially from true commensal ones throughout the town [27], we chose to focus on rural-like species only.

For such a purpose, a Geographic Information System (GIS) of Niamey was implemented from a SPOT satellite image (CNES 2008) using the following seven land cover categories (LCC): Niger river, ponds, bare soils, tarred areas, trees, other greenings and sheet steel-made roofs.

The local urban landscape was described in the vicinity of each of the 11 sampling points where *C*. *gambianus* and/or *A*. *niloticus* specimens were caught (Table 1). To do so, circular buffers of 30 m radius were centered upon each sampling location, and the corresponding landscape was extracted using the R software [38] and the package “raster” [39]. Each circular landscape was described using the percentage of landscape (PLAND) composition metric computed for each LCC [40] using the R package SDMTools [41]. This led to a set of 7 PLAND values (one for each LCC) for each sampling location.

The second step of the analysis consisted in processing these compositional data through a Principal Component Analysis [42] using the R package “ade4” [43]. The first Principal Component (PC1) partly, but highly significantly separated the locations with and without trapped rodents (Monte-Carlo test of between-group inertia, 999 replicates, p = 0.009; [44]). The locations with rodents were associated to high values of PLAND for trees and greenings and low proportion of bare soil and sheet steel-made roofs.

**Fig 2:**
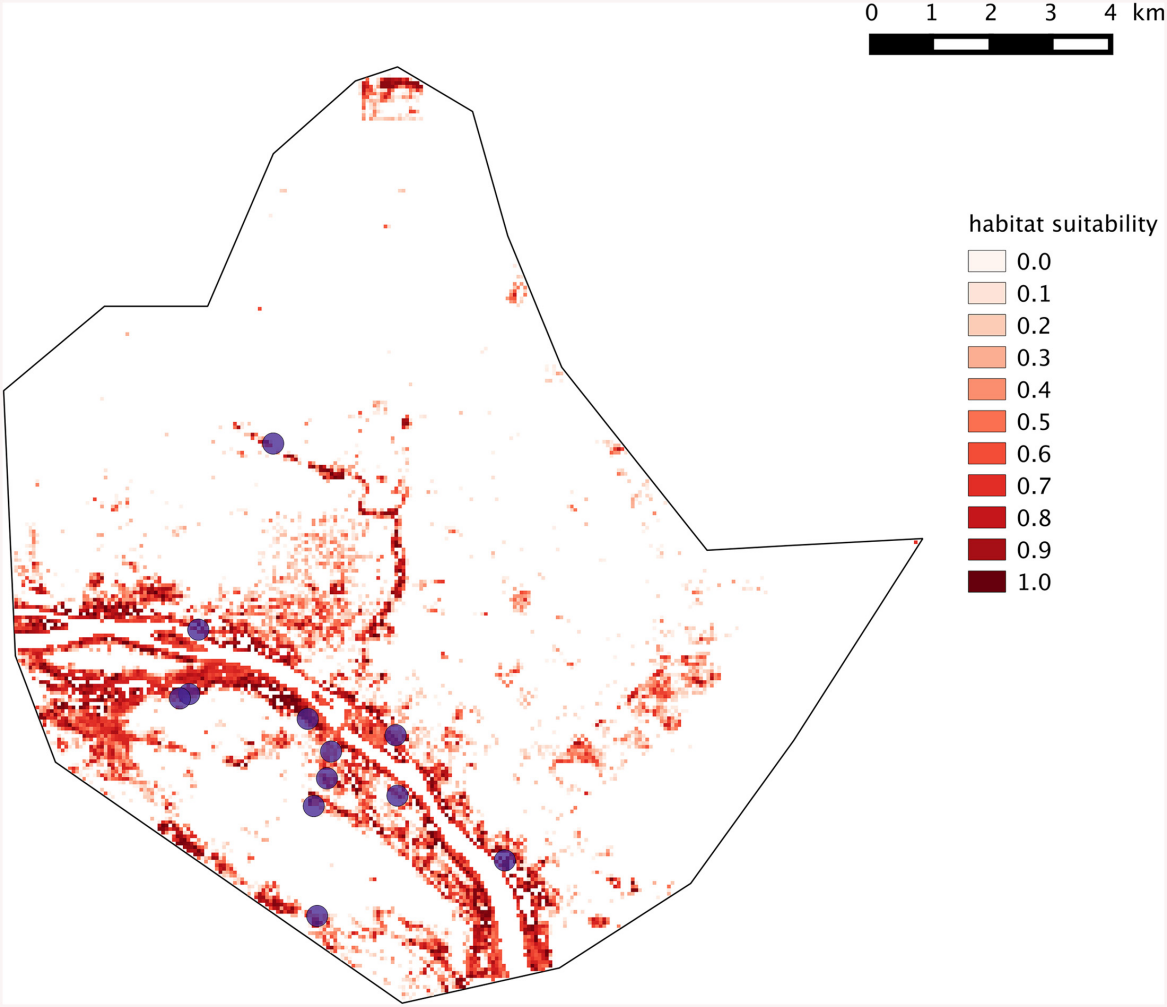
Suitability map of the *Leptospira*-carrying rodent species habitats of across the city of Niamey. Purple dots indicate sites where *A*. *niloticus* and/or *C*. *gambianus* individuals (whatever *Leptospira*-positive or negative) were indeed trapped. White pixels correspond to unsuitable areas where no rodent-borne *Leptospira* is expected.

As a third step, we rasterized the GIS of Niamey into 60x60 meters cells (N = 67,077) within which the percentage of each PLAND was computed. These pixels were then projected onto PC1 as supplementary rows [42]. Their coordinates onto PC1 thus represented their relative position with regards to the gradient of habitat suitability for *Leptospira*-carrying rodent species. The pixel coordinate comprised within the range of the coordinates of locations where *Leptospira*-carrying rodent species were standardized to range between 0 and 1 and subsequently mapped (Fig 2). As such, this map depicts the city-wide spatial variation of the similarity between local habitat and average landscape composition of locations where *Leptospira*-carrying rodent species were caught. In other words, it shows the distribution of suitable habitats for rodent-borne *Leptospira* within Niamey. Expectedly, most of the build-up areas of the city were retrieved as unsuitable for rural-like hence *Leptospira*-carrying rodent species (Fig 2).

## Results

In total, 578 rodents from 49 localities (Fig 1) and five main categories of habitats (i.e., households, markets, coach station, gardens and factories, the latter including a slaughter house, a husking rice industry and an industrial storeroom; Table 1) were investigated for the presence of *Leptospira* using one to four complementary molecular approaches (i.e., metabarcoding, RT-PCR, sequencing, VNTR profiles; Table 1).

First, 550 individuals were screened in triplicated species-specific pools through bacterial 16S metabarcoding (Table 1). A total amount of 287,057 16S sequences was obtained. Among them (which include some bacterial genera of potential medical interest such as *Helicobacter*, *Orientia*, *Mycoplasma*, *Streptococcus*, *Ignatzschineria*), the three replicates of the *A*. *niloticus*-specific pool were found positive for *Leptospira* (3385, 3144 and 3050 *Leptospira* sequences, respectively) while no *Leptospira* sequence were retrieved for the other pools, with the only exception of one *Mastomys*-specific pool in which 37, 58 and 64 *Leptospira* sequences were found. Such a low amount of sequences was intriguing and, after close verifications, we found that that one *Leptospira*-positive *Arvicanthis* individual had been added by error to this slightly positive *Mastomys*-pool (NB: this had been noted on the bench book but this pipetting mistake was then omitted). In order to unambiguously confirm that this lab error was responsible for the few *Leptospira* sequences retrieved within this particular pool, each *Mastomys* individual was screened using the *lipL32* RT-PCR-based procedure: no positive *Mastomys* could be found.

Second, the 50 *Arvicanthis niloticus* specimens from the NGS positive pool as well as 16 additional *A*. *niloticus* and 12 *C*. *gambianus* individuals (that had not been included in the metabarcoding survey) were investigated individually using duplicated *lipL32*-targeting qPCR (Table 1). These 78 animals originated from sites J-LMO1, J-LMO2, J-NOG, J-CYA, J-DAR, J-GAM, J-KIR1 and CRA-3 (Table 1). Among them, seven animals trapped in J-GAM, J-KIR1 and J-LMO2 appeared *Leptospira*-positive twice (with Ct ≤ 31), while one from J-GAM was found positive in only one of the two duplicates (Ct = 38.2). In addition, 12 *Cricetomys gambianus* from sites J-LMO1, J-KIR2, CRA-1 and CRA-2 were also investigated through RT-PCR: one of them (from CRA-2) was found twice *Leptospira*-positive (Ct = 20.3 and 20.5).

Third, the DNA of the seven *A*. *niloticus* and the *C*. *gambianus* qPCR-positive individuals were successfully amplified and sequenced for the *Leptospira rrs* gene (only the *A*. *niloticus* that was qPCR-positive in one of the two duplicates could not be amplified). All eight sequences (Genbank accession numbers KT583752 to KT593759) were found strictly identical (whatever the rodent host species) and, following a Blastn procedure, strictly identical to *L*. *kirschneri* sequences (100% identity; 100% sequence cover; E value = 4.e-^136^; the subsequent most similar sequences belonged to *L*. *interrogans* with 99% identity, 100% sequence cover and E value = 1.e-^134^).

MLVA is a simple and rapid PCR-based method for the identification of most of the serovars of *L*. *interrogans* and *L*. *kirschneri* [37]. MLVA of the VNTR-4, VNTR-7, and VNTR-10 loci were performed in all nine RT-PCR-positive individuals. No PCR product was obtained for the sample that had been found positive in only one out of the two RT-PCR duplicated screenings while two different patterns were retrieved for the remaining ones. First, all *Arvicanthis* samples belonged to genotype I (i.e., VNTR4: 450bp, VNTR7: 320bp, VNTR10: 350bp). Second, genotype II was only represented in the single *Leptospira*-positive *C*. *gambianus* specimen (i.e., VNTR4: 370bp, VNTR7: 320bp, VNTR10: no amplified product). None of these genotypes I and II have been described previously.

All the *Leptospira*-carrying rodents identified in Niamey were trapped in February, October and November. These months all correspond to the dry and cool season. Nevertheless, our sampling did not allow us to investigate seasonality in a satisfying manner, especially within the urban gardens where most rodents were caught in February, October and November, except for one individual trapped in July and two specimens trapped in March.

## Discussion

Our study allows us to highlight for the first time the presence of pathogenic leptospires in Niger. At a wider scale, our data also add to the very rare mentions of *Leptospira* spp. in the Sahel [14], thus confirming that these bacteria do circulate in Sub-Saharan Africa more extensively than currently thought. Moreover, our molecular investigations showed that rodent-borne *Leptospira* in Niamey belonged to *L*. *kirschneri* and to a genotype that had never been identified previously. Its biological features and medical impact, including its virulence in human, remain to be studied in details.

Leptospirosis is one of the most widespread zoonotic diseases around the World. In tropical areas, contact with contaminated water following heavy rainfall and flooding episodes is thought to be a major risk of exposure to pathogenic *Leptospira* spp. [45]. In temperate regions, infection mode is less clear, with recreational water activities and animal caretaking potentially also being of epidemiological importance [4]. In developing countries, high infection rates were also found in cities, essentially within disadvantaged urban areas that usually show poor sanitation and where rodents are numerous (e.g., [17–19, 46, 47]). Here, we point towards a potential other major context of *Leptospira* infection risk in the tropics, namely the market garden areas that surround most cities in developing countries, including those that lie within semi-arid regions.

Indeed, rats are usually considered as the major rodent reservoirs for leptospires worldwide [48]. In Eastern Africa, *Mastomys natalensis* is thought to be the principal source of human infection [49]. *Rattus rattus* and *M*. *natalensis* are from far the most abundant species that were found within Niamey [27]. Yet, out of the 450 specimens of these two species that were tested here, none could be found *Leptospira*-positive. On the contrary, only *Arvicanthis niloticus* and *Cricetomys gambianus* specimens, all trapped within urban market gardens, were detected as carrying *Leptospira*. This strongly suggests that *Leptospira* spp. circulate mostly, if not only in these particular habitats. This is tempting to speculate that irrigated gardens and rice fields along the Niger River provide the warm and moist environmental conditions that favor the bacterium circulation with both the presence of mammalian hosts such as rodents, human-maintained humidity of soils and free water. The absence of rodent-borne leptospires elsewhere in town despite the abundance of potential competent hosts (especially *Rattus rattus* and *Mastomys natalensis*; [27]) as well as poor sanitation conditions would be explained by long-term aridity, thus strongly contrasting with the situation observed in other wetter tropical cities.

The importance of environmental factors in the epidemiology of pathogenic *Leptospira* species has already been suggested in Thailand where the commensal species *Rattus exulans* was found infected much less frequently than other rural / wild species [34]. Ganoza and colleagues [46] further suggested that anthropogenic modification of the urban habitat was a major driver of leptospiral transmission to human. With this in mind, our study emphases the potentially highly critical role of urban market gardening in leptospirosis epidemiology since horticulture rapidly extends within and around towns of most developing countries. In sub-Saharan Africa, these so-called green cities are considered as a trump card to reach the “zero hunger” challenge [50]. For instance, urban and peri-urban horticulture produces most of all leafy vegetables that are consumed in Accra (Ghana), Dakar (Senegal), Bangui (Central African Republic), Brazzaville (Congo), Ibadan (Nigeria), Kinshasa (Democratic Republic of Congo) and Yaoundé (Cameroon), which represent a total population of 22.5 million inhabitants [50]. Yet, the setup of agricultural spaces in close proximity to, when not inside cities or villages raise public health issues since they may favor the maintaining of some pathogenic agents and eventually their vectors or reservoirs, hence potentially increasing the risk of human exposure to the associated diseases, such as malaria (e.g., in Benin: [51, 52]; in Ghana: [53]), various gastro-intestinal infections (e.g., in Benin: [54]) schistosomiasis (e.g., in Ivory Coast: [55, 56] in Niger: [57]), leptospirosis (this study) or potentially toxoplasmosis (e.g., in Niamey, Niger: [58]).

Fine-scale studies show that the impact of these infectious agents may vary at very local scale, depending on the habitat structure and use (e.g., [55, 56]). In the same manner, in Brazilian slums, human cases of leptospirosis seems to aggregate at the very local scale of some households [59], thus suggesting that city-scale studies are inadequate to fully understand the disease epidemiology [48]. These findings, together with our first description of rodent-borne pathogenic *Leptospira* within urban market gardens of Niamey, suggest that investigations are now required in order to (i) provide a more precise picture of *Leptospira* circulation within the urban farming zones of this Sahelian city, and (ii) to look whether human transmission evidence indeed exists in Niger. If this was to be the case, leptospirosis may well represent an important amount of the numerous cases of “fever of unknown origin” that mimic malaria in this semi-arid area. Our GIS-based inferences of suitable areas for *Leptospira*-carrying rodent species in Niamey clearly correspond to intra-city agricultural zones, especially those along the Niger River and the Gountou Yéna wadi (Fig 2). This suggests that human populations at higher risk may well be urban farmers as well as all people that are in close contact with the river waters for their everyday activities (e.g., fishing, clothes and dish washing, bathing, etc). This is the reason why we recommend that investigations about human prevalence are conducted in these areas where leptospires may represent a very impacting though under-diagnosed health issue. Finally, climatic change together with human-mediated modifications of land use accentuates Niger River-associated flooding events (see, for instance, the dramatic episodes that occurred in Niamey in 2010, 2012 and 2013; [60, 61]). From there, we anticipate an increase of leptospirosis’ impact on human health in Niamey in a near future.
